# The Control of Overcrowding in Workhouses

**Published:** 1913-09-27

**Authors:** 


### THE CONTROL OF OVERCROWDING IN WORKHOUSES.

Time after time critics of our Poor-Law system, The Hospital among them, have drawn attention to instances of overcrowding in workhouses, and have demanded reforms which would prevent it. That such overcrowding is attended by the gravest risks to the health of the inmates is obvious. In many cases it cannot be excused even on the grounds of necessity. When one workhouse is overcrowded it is quite common to find that there is plenty of vacant accommodation in the workhouse of a neighbouring Union. In London, for instance, a table prepared by the Local Government Board of the number of inmates of the Metropolitan workhouses and infirmaries on February 10, 1906, showed that in seventeen Unions there was spare accommodation in their workhouses amounting in all to a total of 3,259 beds. On the other hand, the workhouses of fourteen Unions had 3,706 inmates in excess of their registered accommodation. The infirmaries of seventeen Unions had an excess of 1,031 beds, whilst those of eight Unions had deficiencies amounting to 485. By the Poor Law Amendment Act of 1849 Boards of Guardians are given the power to board out any of the inmates of their institutions, when overcrowded, in the institutions of other Boards possessing suitable accommodation. If these powers had been used in 1906 it will be seen from the above figures that there would have been no overcrowding in the London infirmaries, whilst in the workhouses it would have been reduced to very narrow limits. Poor-Law reformers have always felt that where local Boards of Guardians have become so indifferent to their responsibilities as to permit the continued existence of overcrowding the only remedy lay in strong action by the Local Government Board. That such action has not been taken so frequently in the past as was desirable may be readily admitted. Still, by Art. 100 of the General Consolidated Order of 1847, the Guardians were prohibited from admitting to the workhouse a larger number of paupers than that fixed by the Local Government Board, and if such number were exceeded the Clerk was required to report the fact forthwith to that Board. That article, undoubtedly, was a powerful influence in inducing many Boards of Guardians to provide sufficient accommodation in their workhouses. Unfortunately, the Draft Order rescinds this excellent clause.

Art. 3 of the Draft Order which replaces it says nothing at all about overcrowding, but merely lays down that where an institution or a ward shall be appropriated for the reception of a particular class of inmate and the Guardians find it necessary to admit inmates of another class, the Clerk to the Guardians shall forthwith report the facts and the reasons for the Guardians' action to the Local Government Board. If, therefore, the Draft Order is put into operation without amendment Boards of Guardians will be free to overcrowd their institutions and wards, so long as the classification of inmates is adhered to, without any necessity to report the fact of such overcrowding to the Local Government Board.

It is curious to note how great has been the retrogression in this respect since 1842. Art. 11 of the General Order, Workhouse Rules, issued by the Poor-Law Commissioners on February 5, 1842, made it obligatory upon the Guardians after consulting with the medical officer to report to the Commissioners the greatest number of paupers which ought to be admitted to the workhouse, and made it illegal for the Guardians to admit into or retain in the workhouse a larger number. If such number were exceeded a special meeting of the Guardians had to be summoned within seven days to consider the steps necessary to be taken for hiring or otherwise providing additional workhouse accommodation. This article was rescinded in 1847 and the very much milder and less efficient Art. 100 quoted from above substituted for it. Now, in 1913, it is proposed to abolish even the weak safeguards against overcrowding contained in this latter article. Surely Mr. Burns will not allow so retrograde a step to be taken.

